# Low microsatellite instability revisited: a review

**DOI:** 10.1007/s00428-026-04400-w

**Published:** 2026-01-21

**Authors:** Bianca Grosser, Meike Kohlruss, Gisela Keller

**Affiliations:** 1 Institute of Pathology, TUM School of Medicine and Health, Technical University of Munich, Trogerstr. 18, 81675 Munich, Germany; 2 Institute of Pathology and Molecular Diagnostics, University Hospital Augsburg, Augsburg, Germany

**Keywords:** Microsatellite instability, Gastric carcinoma, Colorectal cancer, Dinucleotide repeats, Chemotherapy response, Biomarker

## Abstract

Microsatellite instability (MSI), caused by impaired mismatch repair (MMR), has gained prominence as a biomarker predicting response to immune checkpoint inhibitors in various cancers. MSI-high (MSI-H) tumours exhibit widespread instability across multiple microsatellite loci and are well-characterized. In contrast, low-level microsatellite instability (MSI-L)—marked by instability at a low number of loci—is poorly understood and its biological relevance remains controversial. MSI-L has often been grouped together with microsatellite stable (MSS) tumours, given the lack of consistent molecular distinctions. However, some studies, particularly in colorectal and gastric cancers, have reported that MSI-L correlates with distinct clinical and molecular features, including poorer prognosis, increased tumour mutational burden (TMB) following chemotherapy, and better response to platinum/5-fluorouracil-based neoadjuvant chemotherapy. Notably, these associations frequently involve instability at dinucleotide repeat markers, hinting at a specific subset of MSI-L. Moreover, recent data provide initial evidence that MSI-L may be associated with subtle alterations of genes involved in DNA damage tolerance pathways. This review aims to clarify the current understanding of MSI-L by (a) comparing diagnostic methods and their influence on MSI-L classification, (b) summarizing clinical and molecular associations of MSI-L specifically in gastric and colorectal cancer, (c) highlighting new aspects regarding potential mechanisms underlying MSI-L, focusing on the particular unstable marker and a possible role of the DNA damage tolerance pathways, and (d) discussing whether MSI-L, particularly defined by dinucleotide repeat instability, may serve as a marker for therapeutic vulnerability.

## Introduction

Microsatellite instability (MSI) is characterized by insertions or deletions in repetitive DNA sequences within a tumour, resulting from defects in the mismatch repair (MMR) system. The key players of this system are the mismatch repair genes *MLH1*, *MSH2*, *MSH6*, and *PMS2*. MSI testing plays a critical role in predicting responses to immune checkpoint inhibitors in various cancers, particularly colorectal and gastric carcinomas [1, 2]. MSI in this context is usually characterized by a high level of unstable microsatellite markers in the tumour and is also referred to as high MSI (MSI-H). These tumours are characterized by high mutational burden, distinct molecular profiles, and favourable responses to immunotherapy. In contrast, tumours showing instability at a low number of loci are classified as MSI-low (MSI-L), a category that remains poorly defined and is controversially discussed [3–5].

MSI can be determined by various methods that differ in their accuracy, sensitivity, and application. We believe that the ambiguity surrounding MSI-L arises largely from methodological inconsistencies. Different microsatellite marker panels, cut-off values, and detection platforms (e.g. PCR vs. next-generation sequencing) produce varying results. Furthermore, the lack of a consistent molecular signature has fueled debate on whether MSI-L constitutes a real biological entity or is merely a technical artifact, and MSI-L is now usually grouped together with microsatellite stable (MSS) tumours [6–8]. However, emerging evidence points to potential clinical and biological significance. Some studies in gastric and colorectal cancer link MSI-L—particularly when defined by instability at dinucleotide repeats—to poorer prognosis, altered chemotherapy response, and specific molecular features, such as increased tumour mutational burden post-therapy (Tables 1 and 2).

**Table 1:** Studies reporting differences between MSI-L and MSS tumours regarding survival, clinicopathological features, or molecular alterations in gastrointestinal tumours

Marker panel and MSI-L definition (number of unstable/analysed markers)	Tumours		MSI-L		Relevant associations of MSI-L compared to MSS with			Reference
	Type	***n***	***n*** (%)	Unstable marker	Survival	Clinicopathological features	Molecular alterations	
Bethesda-Panel* + one mononucleotide (BAT40) MSI-L: one of six	AEG I, II, III	363	24 (6.6)	BAT40: 58% Bethesda markers: infrequently	Better cancer-specific survival		Higher CD8 cell count, higher frequency of *TP53* truncating mutations; lower LIN-1 methylation	Imamura et al. 2021 [57]
Bethesda-Panel* MSI-L: one of five	AEG II, III, GC Biopsies before CTx Resected tumours: without CTx after CTx	143 291 326	7 (4.9) 15 (5.2) 13 (4.0)	Dinucleotides: 94%	Worse OS in non CTx tumours	Better response to CTx, intestinal type	Aberrant p53 expression	Kohlruss et al. 2019 [50] Grosser et al. 2020 [66]
Three mononucleotides (BAT25, 26, NR27) + five EMAST markers MSI-L: one of eight	CRC (R0 resected)	159	60 (37.7)	BAT25: most unstable marker	Worse OS	Stage III and IV tumours	EMAST positive tumours	Torshizi Esfahani et al. 2019 [77]
Five mononucleotides MSI-L: one of five	CRC	158	21 (13.3)	Only mononucleotides	Worse OS (stage II)			Nazemal-hosseini Mojarad et al. 2016 [54]
Bethesda-Panel* MSI-L: one of five	CRC	3019	200 (6.6)	Dinucleotides: 97%	Worse OS only in multivariable analysis	Proximal location, larger tumour size		Lee et al. 2015 [53]
Two mononucleotides (BAT26, BAT40) + four dinucleotides + MYCL1 MSI-L: < 40%	CRC	184	22 (12,0)		Tendency of MSI-L with better OS (p = 0.084)		Higher frequency of reduced MGMT expression; lower frequency of COX-2 overexpression	Azzoni et al. 2011 [44]
Bethesda-Panel* MSI-L: one of five	CRC	657	30 (4.6)	BAT25: 20% BAT26:23.2% D2S123: 6.7% D5S346:20.0% D17S250:30.0%		Poor differentiation, mucinous carcinomas, larger tumour size, less lymph node metastasis, less advanced TNM stage		Kim et al. 2009 [58]
Bethesda-Panel* MSI-L: one of five	CRC	940	67 (7.1)	Dinucleotides: 97%		-	Higher frequency of *KRAS* mutation in Dukes’ stage B-D	Asaka et al. 2009 [55]
Bethesda-Panel * + 12 markers MSI-L: < 40% corresponded to one of five of the Bethesda-Panel	CRC	156	18 (11.5)	Dinucleotides: 72%	Worse disease free survival		*KRAS* mutation	Oliart et al. 2006 [52]
Bethesda-Panel* + MYCL1 MSI-L: one unstable dinucleotide or unstable MYCL1	CRC (stage C)	183	51 (27.9)	Only dinucleotides or MYCL1	Worse survival in uni- and multivariable analysis		Higher frequency of loss of *MGMT* expression, lower frequency of *p16* methylation	Kohonen-Corish et al. 2005 [46]
Bethesda-Panel* + two mono-, three di-, one tetranucleotide MSI-L: < 40%	CRC	209	33 (15.8)	Mainly dinucleotides and tetranucleotide	Worse cancer-specific survival in multivariable analysis			Wright et al. 2005 [51]
Fourteen markers MSI-L: < 40%	CRC	172	37 (21.5)			Rarely right sided, low proliferation index		Rudzki et al. 2003 [59]
Bethesda-Panel* + eleven dinucleotides + one tetranucleotide MSI-L: < 40%	CRC early stage: advanced stage:	104 116	53 (51) 30 (25.9)	-			Higher frequency in early and advanced stage; LOH at 1p32, 8p12-22; higher frequency in advanced stage: LOH at 2p16, 7q31, 17q11 and *KRAS* codon 12 mutations	Kambara T et al. 2001 [63]

^*^Bethesda-Panel comprises two mononucleotide (BAT25, BAT26) and three dinucleotide repeats (D2S123, D5S346, D17S250) [10]. Abbreviations: *AEG*, adenocarcinoma of the gastro-oesophageal junction (AEG II and III according to Siewert and Stein) [78]; *CRC*, colorectal cancer; *CTx*, neoadjuvant chemotherapy; *EMAST*, elevated microsatellite instability at selected tetranucleotide repeats; *IHC*, immunohistochemistry; *LOH*, loss of heterozygosity; *MSI-H*, high microsatellite instability; *MSI-L*, low microsatellite instability; *MSS*, microsatellite stable; *TMB*, tumour mutation burden; *TIL*, tumour-infiltrating lymphocyte; *vs.*, versus

**Table 2:** Studies with enriched MSI-L tumour cohorts showing differences compared to MSS tumours

Marker and MSI-L definition (number of unstable/analysed markers)	Tumour type	MSI status	Unstable markers in MSI-L tumours	Method or analysed parameters	Relevant findings regarding MSI-L compared to MSS	Ref
Bethesda-Panel* MSI-L: one of five	AEG II, III, GC	MSS: *n* = 34 (without CTx *n* = 16, with CTx *n* = 18) MSI-L: *n* = 20 (without CTx *n* = 10, with CTx *n* = 10)	Only unstable dinucleotides	Whole-exome sequencing	Higher frequency of variants in the DNA damage tolerance pathway; significant higher TMB in MSI-L vs. MSS tumours with CTx, but not in MSI-L vs. MSS tumours without CTx	Kohlruss et al. 2023 [56]
Bethesda-Panel* MSI-L: one of three dinucleotides	CRC (clinically suspected of HNPCC)	MSS: *n* = 68 MSI-L: *n* = 18 MSI-H: *n* = 12		Family history and clinicopathological features IHC: MLH1, PMS2, MSH2, MSH6	Higher T-grade and lower antitumour immune response (lack of intra-epithelial, peri- and intertumoural lymphocyts and Crohns’s-like lymphoid reaction) in MSI-L vs. MSS; no difference in family history of MSI-L vs. MSS; normal expression of MLH1, MSH2, MSH6, PMS2 in MSI-L	Kets et al. 2006 [45]
Bethesda-Panel* + one mononucleotide (BAT40) + five dinucleotides + one tetranucleotide MSI-L: one to < 30%	CRC	MSS: *n* = 15 MSI-L: *n* = 14, MSI-H: *n* = 12	Only unstable di- or tetra-nucleotides	cDNA microarray and principal component analysis; clinicopathological features	MSI-L significant different from MSS and MSI-H for principal component 10 designated the MSI-L separator; no difference of clinicopathological features between MSI-L and MSS	Mori et al. 2003 [65]
Six markers (BAT26, BAT40, AT3, D2S123, F13B, MYCL) MSI-L: one or two of six	CRC	MSS: *n* = 40 MSI-L: *n* = 30 MSI-H: *n* = 32		Immunohistochemical analysis of TILs: (CD3,CD8) and apoptosis rate	Intermediate values for TILs and apoptosis rate of MSI-L	Michael-Robinson et al. 2001 [61]
Bethesda-Panel* + MYCL MSI-L: one of five or/and unstable MYCL, but no unstable mononucleotide	CRC	MSS: *n* = 23 MSI-L: *n* = 44 MSI-H: *n* = 23	D2S123: 24% MYCL: 67%	*MGMT* methylation *KRAS* mutation	Highest frequency of *MGMT* silencing and *KRAS* mutations in MSI-L	Whitehall et al. 2001 [64]
Six markers (BAT26, BAT40, AT3, D2S123, F13B, MYCL) MSI-L: one or two of six	CRC	MSS: *n* = 51 MSI-L: *n* = 38 MSI-H: *n* = 25		*KRAS* mutation, LOH 5q, 17p, 18q IHC: b-catenin, p53	Higher frequency of *KRAS* mutations and lower frequency of LOH 5q in MSI-L vs. MSS. Lower frequency of concordance of aberrant b-catenin expression and LOH 5 q in MSI-L vs. MSS	Jass et al. 1999 [62]

^*^Bethesda-Panel comprises two mononucleotide (BAT25, BAT26) and three dinucleotide repeats (D2S123, D5S346, D17S250) [10]. Abbreviations: *AEG*, adenocarcinoma of the gastro-oesophageal junction (AEG II and III according to Siewert and Stein) [77]; *CRC*, colorectal cancer; *CTx*, neoadjuvant chemotherapy; *HNPCC*, hereditary non polyposis colorectal cancer; *IHC*, immunohistochemistry; *LOH*, loss of heterozygosity; *MSI-H*, high microsatellite instability; *MSI-L*, low microsatellite instability; *MSS*, microsatellite stable; *TMB*, tumour mutation burden; *TIL*, tumour-infiltrating lymphocyte; *vs.*, versus

This review aims to clarify the current understanding of MSI-L by (a) comparing diagnostic methods and their influence on MSI-L classification, (b) summarizing clinical and molecular associations of MSI-L specifically in gastric and colorectal cancer, (c) highlighting new aspects regarding potential mechanisms underlying MSI-L, focusing on the particular unstable marker and a possible role of DNA damage tolerance pathways, and (d) discussing whether MSI-L, particularly defined by dinucleotide repeat instability, may serve as a marker for therapeutic vulnerability.

Through this structured analysis, we aim to determine whether MSI-L is a distinct biologic subgroup or simply a diagnostic grey zone.

## Methods for MSI determination and MSI-L definition

### Polymerase chain reaction (PCR)–based assay

Polymerase chain reaction (PCR)–based MSI analysis is one of the most widely used and validated methods for detecting MSI in tumours. The traditional approach for PCR-based testing relies on amplifying specific microsatellite markers using fluorescently labelled primers and subsequently analysing fragment length differences in tumour and normal DNA samples through capillary electrophoresis [9].

The most commonly used marker panel for MSI testing is the National Cancer Institute (NCI)-endorsed Bethesda-Panel, which consists of five microsatellite markers: BAT25, BAT26, D5S346, D2S123, and D17S250 [10]. These markers include both mononucleotide (BAT25 and BAT26) and dinucleotide repeats (D5S346, D2S123, and D17S250). Tumours are classified as MSI-H if two or more markers show instability, as MSI-L if only one marker is unstable, and as MSS if no markers are unstable [10–12]. If only one marker is unstable, an analysis of five additional markers, the so-called secondary panel, is recommended, including one mononucleotide, three dinucleotides, and one tetranucleotide repeat with a cut-off ≥ 30% unstable marker to define a tumour as MSI-H [13, 14]. If various microsatellite markers and numbers are analysed, a cut-off value of ≥ 30–40% unstable markers is considered an MSI-H tumour, < 30–40% an MSI-L tumour, and no unstable markers an MSS tumour (Table 1) [11, 12].

Over the years, refinements in PCR-based MSI testing have enhanced sensitivity and specificity, notably through multiplex PCR and the use of monomorphic mononucleotide repeats, which outperform dinucleotide markers in MSI-H detection in colorectal tumours [15–17]. The pentaplex MSI-PCR assay, using five mononucleotide markers (BAT25, BAT26, NR-21, NR-24, MONO-27), is now widely recommended particularly for assessing immunotherapy eligibility [15, 18, 19].

Despite the high sensitivity and specificity of the mononucleotide-based assay for determining MSI-H in colorectal cancer, the performance may vary depending on the tumour type, and an increase in false negative results has been reported, particularly in endometrial carcinomas. MSI in these tumours is often characterised by complex and more subtle microsatellite shifts that are more difficult to identify and has been described in context with unusual expression patterns of MMR proteins [20, 21]. Assays have therefore been developed that analyse a larger number of mononucleotide markers or longer mononucleotide repeat markers (LMR-MSI assay, Promega) [22]. A real-time PCR–based test system for analysing seven monomorphic homopolymer biomarkers has been developed as a rapid screening tool for detecting MSI-H in colorectal cancer (Idylla system, Biocartis). However, the system does not detect MSI-L, since cases with a single unstable marker are categorized as microsatellite stable, and reduced sensitivity has been observed in non-gastrointestinal tumour types [23, 24]. A comparative study of four different PCR-based test systems for endometrial and colorectal cancer showed a high degree of concordance in MSI-H determination only after a re-evaluation of initially inconsistently classified cases, highlighting the importance of tumour-specific assay validation and testing by well-trained users for the Promega and Bethesda-Panels, as well as the need for larger amounts of tumour tissue, especially for the Idylla system [25].

The assay systems currently used in diagnostics were recently summarised by Dietmaier et al. [14]. Additionally, because the focus of our review is MSI-L, we emphasise that current methods are optimized for MSI-H detection and that MSI-L classification varies depending on the marker concept (mononucleotide only versus mixed panels), potentially identifying biologically distinct tumour groups [18, 19].

### Next-generation sequencing (NGS)–based assay

Next-generation sequencing (NGS)–based MSI detection represents a more advanced and comprehensive approach to assess MSI. NGS-based MSI detection offers a comprehensive alternative by analysing thousands of microsatellite loci genome-wide, enabling higher sensitivity and precision—especially in tumour types where PCR-based assays may underperform. It compares microsatellite length distributions in tumour DNA to a reference, identifying instability via computational algorithms [19].

Several complex bioinformatics tools support this analysis, including MOSAIC, mSINGS, MSIsensor, and MANTIS, each of which utilizes different computational approaches to assess microsatellite stability [7, 26–29]. One of the key advantages of NGS-based MSI testing is its ability to provide a quantitative MSI score, enabling the detection of borderline cases that may be missed by categorical PCR-based classification. Some MSI-intermediate (MSI-I) cases may align with MSI-L in PCR-based assays, but essentially, the specific algorithms have been validated and optimized to detect MSI-H [19, 29]. A key advantage is the integration of this MSI analysis into routine tumour sequencing workflows, allowing simultaneous detection of other genomic alterations such as tumour mutation burden (TMB), oncogenic drivers, and MMR gene mutations. While highly sensitive and specific, most algorithms were developed for colorectal tumours and may require calibration for other entities.

Targeted gene panels enable molecular tumour profiling, including MSI-H detection; however, further standardization of marker selection and cut-off thresholds is required to ensure reliable NGS-based MSI-H assessment in routine clinical practice [14]. Then, as sequencing becomes more accessible and bioinformatics tools advance, NGS-based MSI detection is poised to become standard in precision oncology [19].

### Immunohistochemistry

Immunohistochemistry (IHC) for MMR proteins is a widely used method for detecting MSI-H, offering insight into which MMR gene (*MLH1*, *MSH2*, *MSH6*, *PMS2*) may be defective by assessing protein expression in tumour tissue. Loss of staining indicates MMR deficiency, supporting MSI-H classification and informing treatment decisions and Lynch syndrome screening [18, 19]. As the MMR protein MLH1 binds to PMS2 and MSH2 binds to MSH6, the loss of one of these heterodimer complexes leads to the classic staining pattern that indicates MMR deficiency and shows a strong correlation in the range of 93% to 99% with MSI-H status in gastrointestinal tumours [30]. Aberrant staining patterns, e.g. isolated loss of MSH6 or PMS2 or reduced or focal expression, have been reported and should be interpreted in conjunction with PCR-based MSI analysis. A detailed description of these staining patterns can be found in the cited studies and review articles [18, 21, 30].

However, IHC is limited in identifying MSI-L. Some studies report reduced expression of MSH6 or MSH3 in MSI-L tumours, but findings remain inconsistent, particularly in gastric cancer [31–33]. Loss of MSH6 may be associated with both MSI-L and MSI-H phenotypes [18, 19]. Thus, to date, no reliable IHC marker exists for detecting MSI-L.

## Clinicopathological and molecular features of MSI-L compared to MSS tumours

### MSI-L and MSS tumours are largely similar

Many studies comparing MSI-L and MSS tumours—especially in colorectal and gastric cancers—have reported no significant differences in clinical presentation, prognosis, or molecular characteristics [7, 34–43]. These findings support the current clinical practice of grouping MSI-L with MSS tumours.

Regarding the methodology used to identify and define MSI-L in these studies, most used the Bethesda-Panel, which includes two mononucleotide and three dinucleotide repeats, or similar PCR-based assays [39, 40]. Other studies, particularly those focused on colorectal carcinomas, analysed additional or alternative markers and similarly, as mentioned, found no specific differences between MSI-L and MSS tumours [34–37]. Of particular note is a study by Laiho et al. [38], in which 90 BAT26-stable colorectal carcinomas were analysed using 377 additional markers. Various cut-off values for defining MSI-L were tested, but no significant differences in tumour stage, grade, or location between MSI-L and MSS cases were identified. Thus, the authors concluded that MSS and MSI-L tumours likely share a common molecular background and that dividing them into separate groups is not justified [38].

Similarly, alterations in *KRAS*, *BRAF*, or *MGMT*, as well as promoter methylation and loss of heterozygosity (LOH), did not distinguish the two groups [36–38, 40].

Given the involvement of the mismatch repair genes *MLH1*,* MSH2*,* PMS2*, and *MSH6* in the MSI-H phenotype, expression analysis of these genes was of particular interest, but no differences were found between MSI-L and MSS tumours in this regard [44–47]. Considering a possible role of *MSH6* in the MSI-L phenotype, screening for mutations in this gene has been performed, though somatic or germline variants have only rarely been detected [47].

Studies based on NGS technology, which analyse a large number of microsatellite sequences using various bioinformatic algorithms to determine MSI, have also failed to identify meaningful distinctions between MSI-L and MSS cases as determined by standard assays. For example, Hause et al. [7] analysed over five million microsatellite (MS) loci—primarily mononucleotide repeats—and found no difference in the number of additional MS alleles in the tumours compared to normal tissue, nor in the total number of unstable microsatellites [7]. Similarly, other studies reported no significant differences in the number of MSI events between MSI-L and MSS tumours [41, 42].

Based on this body of evidence, MSI-L, when reported at all, and MSS tumours are now generally considered one group. These consistent results led to guidelines, such as those from the European Society for Medical Oncology (ESMO), recommending the abandonment of the MSI-L category and its classification as MSS [48]. Furthermore, recent research using whole-exome or genome-wide strategies to characterize MSI does not recognize MSI-L as a distinct category [49]. Nonetheless, this generalization is challenged by studies that identify specific features in MSI-L tumours when stratified by marker type or treatment context. We are aware that a considerable number of these studies were published several years ago, but we believe that they deserve special attention (Tables 1 and 2). They will be discussed in detail in the following section, with a focus on specific unstable microsatellite markers where such information is available.

### MSI-L as a distinct subgroup: emerging evidence

Contrary to the mainstream view, a subset of studies in gastrointestinal tumours suggests that MSI-L may represent a biologically distinct group, especially when defined by instability at dinucleotide repeats. The studies reporting differences between MSI-L and MSS tumours in unselected patient cohorts or specific subgroups are summarized in Table 1. Due to the relatively low incidence of MSI-L, several investigations enriched their cohorts by selecting tumours based on MSI status to increase the number of MSI-L tumours. These studies are summarized in Table 2.

#### Distinct clinicopathological features and prognosis in MSI-L tumours

Several studies have reported an association of MSI-L with adverse clinical outcomes in gastric or colorectal cancer, either in univariable or multivariable analyses [46, 50–54]. In a cohort of 940 colorectal cancer patients (Dukes’ stage B–D), patients with MSI-L tumours had an intermediate prognosis, with worse outcomes than MSI-H cases but better than MSS tumours [55]. This gradation suggests that MSI-L may represent a biologically meaningful intermediate phenotype. Most of these studies determined MSI status using the Bethesda-Panel, sometimes supplemented with additional markers, particularly tetranucleotide repeats. Notably, in studies specifying the marker-specific instability, dinucleotide markers were predominantly affected [46, 50, 51, 53, 55]. In a more detailed analysis by our group on the MSI-L instability pattern in gastric carcinomas, 94% of dinucleotide repeat instabilities were caused by the insertion of two base pairs [56]. Importantly, four of the five studies that observed a predominance of dinucleotide instability and analysed the prognostic relevance of MSI-L found an association with worse cancer-specific or overall survival [46, 50, 51, 53, 55]. One study utilizing a panel of five mononucleotides for MSI determination also reported that MSI-L status correlated with poor survival in a subgroup of stage II colon cancer patients [54].

In contrast, a study of gastroesophageal junction carcinomas found a positive correlation between MSI-L and improved survival [57]. In that study, MSI-L was primarily based on instability at the mononucleotide repeat BAT 40, which was tested in addition to the Bethesda-Panel. Another analysis of 184 colorectal cancers, using seven microsatellite markers (including two from the Bethesda-Panel), also found a trend towards better survival in MSI-L tumours [44].

MSI-L tumours have also been linked to specific histopathological features. In gastric carcinoma, MSI-L was associated with the intestinal histotype [50]. In colorectal cancers, MSI-L was more frequently found in tumours with mucinous differentiation, poor differentiation, larger tumour size, and lower incidence of lymph node metastases [53, 58]. Regarding tumour location, conflicting results as a preferential proximal (right-sided) and preferential left-sided location have been reported [53, 59].

In relation to response to therapy, an association of MSI-L with a good response to platinum/5-fluorouracil (5-FU)–based neoadjuvant chemotherapy has been demonstrated for gastric cancer [50]. In locally advanced rectal cancer, MSI-L status predicted a lower rate of pathological complete response after neoadjuvant chemoradiation [60] highlighting its potential predictive value, which may differ depending on tumour type and the applied therapy regimen.

Additionally, tumour-infiltrating lymphocyte (TIL) counts and apoptotic indices in MSI-L tumours were found to be intermediate between MSI-H and MSS, suggesting partial immunogenicity [61]. In a study of familial colorectal cancers, MSI-L tumours exhibited significantly reduced immune cell infiltration and more advanced tumour stages, along with a tendency towards lymph node positivity. This study used the classical Bethesda-Panel, and MSI-L was defined by instability at a dinucleotide marker. The authors further noted that 78% of instabilities were due to the addition of a single dinucleotide repeat [45]. These findings support the view that MSI-L tumours, while lacking the strong immunogenic signature of MSI-H tumours, may still differ substantially from MSS tumours in their biological behaviour.

#### Distinct molecular features in MSI-L tumours

Molecular alterations were compared between MSI-L and MSS tumours in both unselected and selected tumour series and are included in Tables 1 and 2, respectively.

Several molecular differences have been noted. A commonly reported finding is a higher frequency of *KRAS* mutations in MSI-L tumours or in specific tumour subgroups [52, 55, 62, 63]. However, this result was not consistently observed, with other studies failing to confirm the association [36–38, 40]. In a comparative analysis of 25 MSI-H, 38 MSI-L, and 51 MSS-selected colorectal carcinomas, *KRAS* mutations, LOH on chromosomes 5q, 17p, and 18q, and the expression of p53 and beta-catenin were examined, with a significantly higher frequency of *KRAS* mutations and a lower frequency of LOH on 5q found for the MSI-L tumours. Based on these results, the authors suggested that MSI-L tumours represent a distinct group possibly characterized by a suppressor/mild-mutator phenotype [62].

Also notable are studies reporting frequent loss of MGMT expression in colorectal carcinomas [46, 64]. Whitehall et al. [64] demonstrated *MGMT* silencing by promotor methylation, with the highest frequency observed in MSI-L tumours. In the same cohort, methylated MSI-L tumours frequently harboured *KRAS* mutations, supporting a molecular association between these alterations. Mori et al. [65] performed a cDNA microarray and principal component analysis on 12 MSI-H, 14 MSI-L, and 15 MSS colorectal carcinomas and identified a specific component—termed the “MSI-L separator”—distinguishing MSI-L from MSI-H and MSS tumours. Among the differentially expressed genes, reduced *MGMT* expression was again observed in the MSI-L group. Of note, MSI-L status in this study was defined exclusively by instability at di- or tetranucleotide repeats. The authors also noted that the molecular effect of MSI-L was more subtle than that of MSI-H, potentially explaining the similar clinical features between MSI-L and MSS patients [65]. Thus, *MGMT* silencing and *KRAS* mutations appear to be recurrent molecular alterations in MSI-L tumours. Regarding the tumour suppressor gene *TP53*, aberrant p53 expression and a higher frequency of *TP53* truncation mutations have been reported in tumours of the stomach and esophagogastric junction [57, 66].

Aberrant protein expression of the *MSH3* gene or frequent LOH at the *MSH3* locus has been observed and implicated in the aetiology of MSI-L in colon carcinomas [31, 32]. *MSH3* germline mutations were described in patients with adenomatous polyposis, and an elevated microsatellite instability at selected tetranucleotide repeats, called EMAST, was found in some of these tumours [67]. A partial overlap of EMAST with MSI-L and also with MSI-H has been reported in colorectal cancer [68]. In a study on gastric carcinomas, however, no obvious overlap of EMAST with MSI-L and no significant association of MSH3 expression with MSI-H, MSI-L, and MSS were found [33].

In gastric carcinomas, using whole-exome sequencing, our group compared various DNA repair and DNA damage tolerance pathways between MSS and MSI-L tumours [56]. Although not statistically significant, a striking difference was a higher frequency of DNA variants in the DNA damage tolerance pathways in the MSI-L group (35%) compared to the MSS group (18%). The MSI-L tumours also showed a higher tumour mutation burden (TMB) after platinum/5-FU-based chemotherapy, supporting their increased chemosensitivity. Notably, these MSI-L cases were characterized by dinucleotide repeat instability [56]. Of note, this observation supports our previous findings on pre-therapeutic tumour biopsies, which showed a better response to neoadjuvant CTx in patients with MSI-L tumours [50]. Together with these findings, we obtained initial indication that MSI-L tumours may exhibit subtle alterations in the DNA damage tolerance pathway, which could contribute to their distinct molecular and therapeutic profile.

Overall, although most studies support grouping MSI-L with MSS tumours, a subset of data suggests that MSI-L—when defined by dinucleotide instability—may reflect a specific phenotype with prognostic and therapeutic relevance. These findings underscore the importance of marker-specific analysis and support re-evaluation of MSI-L as more than a diagnostic artifact.

## Microsatellite marker-specific characteristics and functional implications of MSI-L

The conflicting data on MSI-L is probably primarily due to the heterogeneity of the microsatellite markers used for MSI testing. In particular, whether mononucleotide or dinucleotide repeats are included in the panel profoundly influences which tumours are classified as MSI-L. Studies consistently show that MSI-L tumours often demonstrate instability primarily at dinucleotide markers. For example, analyses based on the Bethesda-Panel—which includes both mono- and dinucleotide repeats—reveal that dinucleotide markers (D2S123, D17S250, D5S346) are most frequently unstable in MSI-L tumours [15, 39, 43, 50, 51, 53, 55, 56, 58]. In contrast, panels relying solely on mononucleotide markers (e.g. the Pentaplex assay) probably identify different tumour populations under the same label [15, 45, 56].

Detailed studies have shown that instability in dinucleotide markers in MSI-L tumours frequently involves small insertions, typically the addition of one or two repeat units [45, 56]. For instance, Kohlruss et al. reported that 94% of observed dinucleotide instabilities in MSI-L gastric cancers were insertions of exactly two base pairs [56]. These subtle mutations may reflect a unique underlying mechanism, distinct from the more pronounced genomic instability seen in MSI-H tumours.

**Fig. 1:**
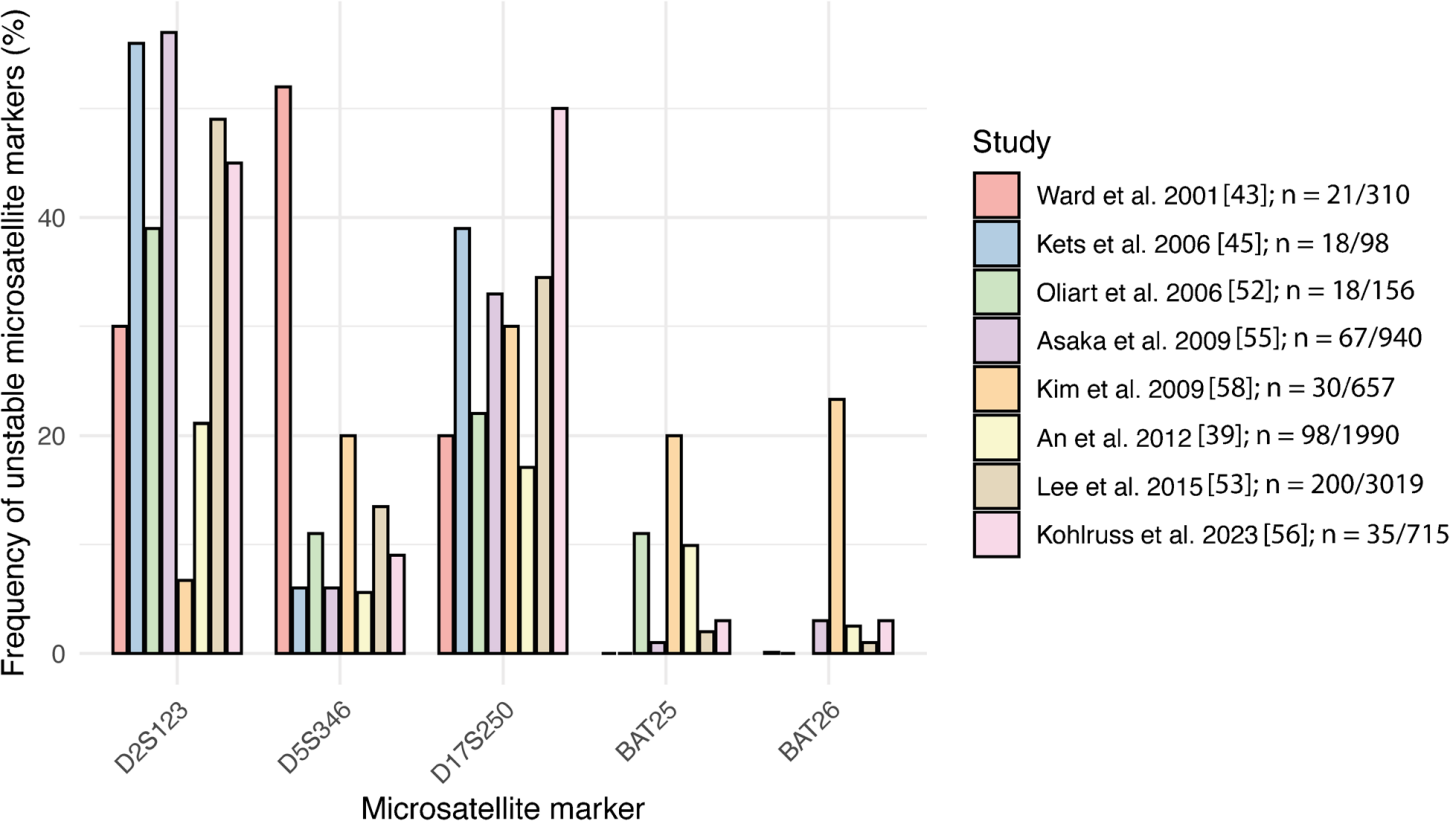
Frequency of the unstable microsatellite markers of the Bethesda-Panel in different studies. The frequency of the unstable microsatellite markers of the Bethesda-Panel encompassing two mononucleotide (BAT25, BAT26) and three dinucleotide markers (D2S123, D5S346, D17S250) is shown for different studies providing this information. The absolute number (*n*) of MSI-L/analysed tumours in each study is indicated

Interestingly, the frequency and mutation spectrum of individual markers also vary. In most studies reporting these data, D2S123 is the most frequently altered marker, followed by D17S250 and D5S346, indicating that its repeat structure might be particularly prone to replication stress. An overview of instability frequencies per marker of the Bethesda-Panel and the total number of tumours analysed in these studies is shown in Fig. 1. These findings suggest that the quality and specific characteristics of each marker play a significant role.

**Fig. 2:**
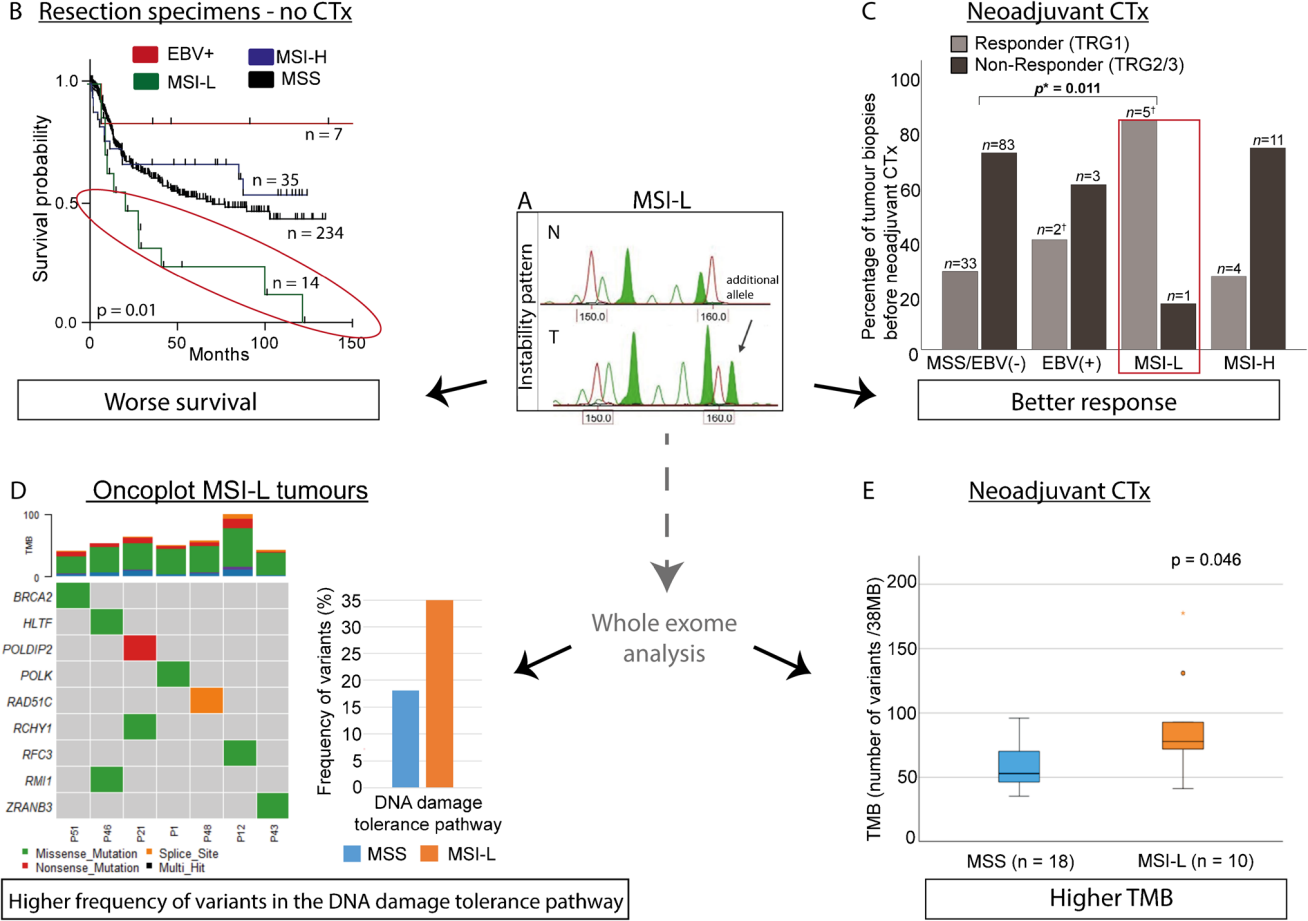
Summary of the most important results of our studies on MSI-L in gastric carcinomas [50, 56]. **A** An example of a typical pattern of MSI-L at marker D17S250 in capillary electrophoresis is shown [56]. An additional allele in the tumour (T) in comparison to the pattern in the corresponding non-tumorous tissue (N) is indicated by an arrow. The green peaks are the microsatellite alleles; the red peaks are internal size standards. The x-axis displays the size in bases and the y-axis the respective fluorescence intensity. **B** MSI-L and worse survival in patients not treated with neoadjuvant CTx. Kaplan–Meier survival curves of MSI-L, MSI-H, Epstein-Barr virus (EBV) (+), and MSS/EBV(-) in resected tumour specimens of overall 290 patients not treated with neoadjuvant CTx are shown [50]. According to the molecular classification of gastric cancer published by the Cancer Genome Atlas Network (TCGA) [6], EBV positive tumours were considered a separate group in that study. The MSI-L group (indicated by a red circle) shows the worst survival. *p* value refers to the log rank test (overall). **C** MSI-L and better response to neoadjuvant CTx. Response to neoadjuvant CTx of MSI-L, in comparison with MSI-H, EBV(+), and MSS/EBV(-) of 142 pre-therapeutic tumour biopsies is shown [50]. MSI-L tumour biopsies show a significantly higher frequency of responding patients. *p* values refer to the chi-square test, each compared with MSS/EBV(-). Response was determined histopathologically and was classified into three tumour regression grades (TRG) according to Becker et al. [76]. Patients with TRG1 (< 10% residual tumour cells/tumour bed) were classified as responders. Patients with TRG 2,3 (10–50 and > 50% residual tumour cells/tumour bed) were classified as non-responders. **D** MSI-L tumours show a higher frequency of DNA variants in the DNA damage tolerance pathway [56]. The frequency of MSI-L and MSS tumours with sequence variants is shown. The oncoplot of MSI-L tumours illustrates which genes have sequencing variants. **E** MSI-L tumours show a higher tumour mutation burden (TMB) after neoadjuvant CTx [56]. The median TMB of MSI-L tumours was significantly higher compared to MSS tumours after CTx. No difference was observed for tumours not treated with CTx (figure not included here). *p* value: Mann–Whitney U test. Abbreviations: EBV, Epstein-Barr virus; MSI-L, low microsatellite instability; MSI-H, high microsatellite instability; CTx, chemotherapy; TMB, tumour mutation burden; TRG, tumour regression grade

Research on microsatellite evolution has shown that expansions among dinucleotide alleles occur more frequently than contractions, and that mutation rates of individual microsatellite markers are highly heterogeneous [69]. This heterogeneity appears to be mirrored in tumour biology. The variability in mutation rates is believed to stem from intrinsic properties of the repeat sequences themselves, including repeat motif, length, and number of repeat units [69–71]. Importantly, certain microsatellites may form secondary DNA structures that interfere with DNA replication, leading to a temporary stalling of the DNA replication fork. To resume replication, cells must activate complex DNA damage tolerance pathways, involving replication fork remodelling and restart mechanisms [72–74]. This supports our recent findings that MSI-L tumours, particularly those with unstable dinucleotide markers, may be associated with alterations in DNA damage tolerance pathways. These pathways are essential for resolving replication fork stress and maintaining genomic stability under genotoxic conditions. Given that platinum-based chemotherapy agents such as cisplatin are also known to induce replication stress by stalling DNA replication forks, it is plausible that MSI-L tumours with impaired DNA damage tolerance mechanisms exhibit increased chemosensitivity [74]. This hypothesis is supported by our studies on pre-therapeutic tumour biopsies showing better response rates and higher tumour mutational burden in MSI-L tumours post-chemotherapy, but worse prognosis of patients with primarily resection of the tumour and no CTx treatment [50, 56]. A summary of the main findings of our studies is shown in Fig. 2.

Therefore, we propose that MSI-L, defined by unstable dinucleotide markers in the Bethesda-Panel—especially D2S123 and D17S250, which show the highest instability rates—may reflect a dysfunction of the DNA damage tolerance pathways and represents a unique biological entity. This subset may possess a vulnerability to replication stress-inducing therapies, suggesting its potential as a predictive biomarker and therapeutic target.

## Conclusion and future perspectives

MSI-L defines a heterogeneous category that encompasses biologically diverse tumours. Marker composition strongly influences MSI-L classification. According to current diagnostic guidelines, when MSI-L is detected or suspected, analysis with a secondary marker panel should be performed and interpreted in combination with immunohistochemical results.

Instability at dinucleotide repeats, especially D2S123 and D17S250, may define a subset of MSI-L with unique molecular and clinical characteristics. This type of MSI-L may indicate a dysfunction of the DNA damage tolerance pathways and deserves intensive further research.

Similar to studies that define specific mutation signatures using cell line models with dysfunctional mismatch repair and replicative polymerase genes [75], studies on genes involved in the DNA damage tolerance pathways could provide mechanistic insights into the occurrence of MSI-L and, beyond that, may characterise a genome-wide mutation signature with biological relevance.

In addition, future research should focus on systematically analysing the DNA sequences of microsatellite markers for their potential to form secondary structures and induce replication stress. In this context, whole-exome or whole-genome analysis focusing on dinucleotide repeat microsatellite markers in combination with mutation analysis of genes involved in DNA damage tolerance pathways may be helpful. This could clarify the functional properties of specific microsatellite markers and their interactions with DNA replication stress pathways to better define the MSI-L phenotype and its therapeutic implications.
